# Aortic valve laceration following rotational atherectomy: a case report

**DOI:** 10.1093/ehjcr/ytae226

**Published:** 2024-05-09

**Authors:** Farrah Othman, Gerald Yong, Alan Whelan, Abdul Rahman Ihdayhid

**Affiliations:** Department of Cardiology, Fiona Stanley Hospital, 11 Robin Warren Drive, Murdoch, Perth, WA 6150, Australia; Department of Cardiology, Fiona Stanley Hospital, 11 Robin Warren Drive, Murdoch, Perth, WA 6150, Australia; Department of Cardiology, Fiona Stanley Hospital, 11 Robin Warren Drive, Murdoch, Perth, WA 6150, Australia; Department of Cardiology, Fiona Stanley Hospital, 11 Robin Warren Drive, Murdoch, Perth, WA 6150, Australia; Cardiovascular Research and Innovation Laboratory, Harry Perkins Institute of Medical Research, Perth, Australia; Curtin Medical School, Curtin University, Perth, Australia; UWA Medical School, University of Western Australia, Perth, Australia

**Keywords:** Rotational atherectomy, Iatrogenic aortic valve laceration, TAVR for native pure aortic regurgitation, Case report, Complication

## Abstract

**Background:**

Iatrogenic aortic valve injury during cardiovascular catheterization interventions is extremely rare. Severe aortic regurgitation that ensues can be catastrophic and the management is typically with surgical valve replacement or repair. Percutaneous management of native pure aortic regurgitation is difficult due to anatomical challenges and the limitations of current transcatheter heart valve technology to anchor in the absence of leaflet or annular calcification.

**Case Summary:**

An 82-year-old female underwent rotational atherectomy (RA) for a severely calcified stenosis of the left anterior descending artery. The patient was discharged well following placement of two drug eluting stents. She represented to hospital 7 days later with acute pulmonary oedema. Bedside transthoracic echocardiography demonstrated new, severe AR with preserved left ventricular size and function. Review of the prior percutaneous coronary intervention revealed significant trauma to the aortic valve during RA, with contrast seen refluxing into the LV during diastole, evolving throughout the procedure. Given the patient was not an operative candidate, an oversized transcatheter aortic valve was successfully implanted. In the post-operative setting, the patient suffered a stroke. Extensive hypoattenuated leaflet thickening (HALT) and thrombus was seen on dedicated 4D CT imaging. She made full neurological recovery and valve function returned to normal following a period of anticoagulation.

**Conclusion:**

Although iatrogenic aortic valve laceration is rare, this case highlights several important learning points including the importance of good guide catheter support during RA; the feasibility of Transcatheter Aortic Valve Replacement for pure native AR; and the detection and management of HALT.

**Learning points**

- During rotational atherectomy, good guide engagement is paramount when approaching severely stenosed, calcified, and distal coronary lesions.
- Transcatheter aortic valve replacement (TAVR) for native pure aortic regurgitation is feasible using a significantly oversized self-expanding valve.
- Post-TAVR stroke should prompt clinicians to investigate for hypoattenuated leaflet thickening with a dedicated cardiac CT, as early diagnosis and treatment are associated with good outcomes.

## Introduction

Iatrogenic aortic valve injury during cardiovascular catheterization interventions is extremely rare, with very few documented case reports. To the best of our knowledge, it has not been documented after rotational atherectomy (RA).^1,2^ The present case describes traumatic aortic valve injury following RA with subsequent severe aortic regurgitation (AR) requiring treatment with an oversized transcatheter aortic valve. Although iatrogenic aortic valve laceration is rare, this case highlights several important learning points including the importance of good guide catheter support during RA; the feasibility of transcatheter aortic valve replacement (TAVR) for pure native AR; and the detection and management of hypoattenuated leaflet thickening (HALT).

## Summary figure

**Figure:**
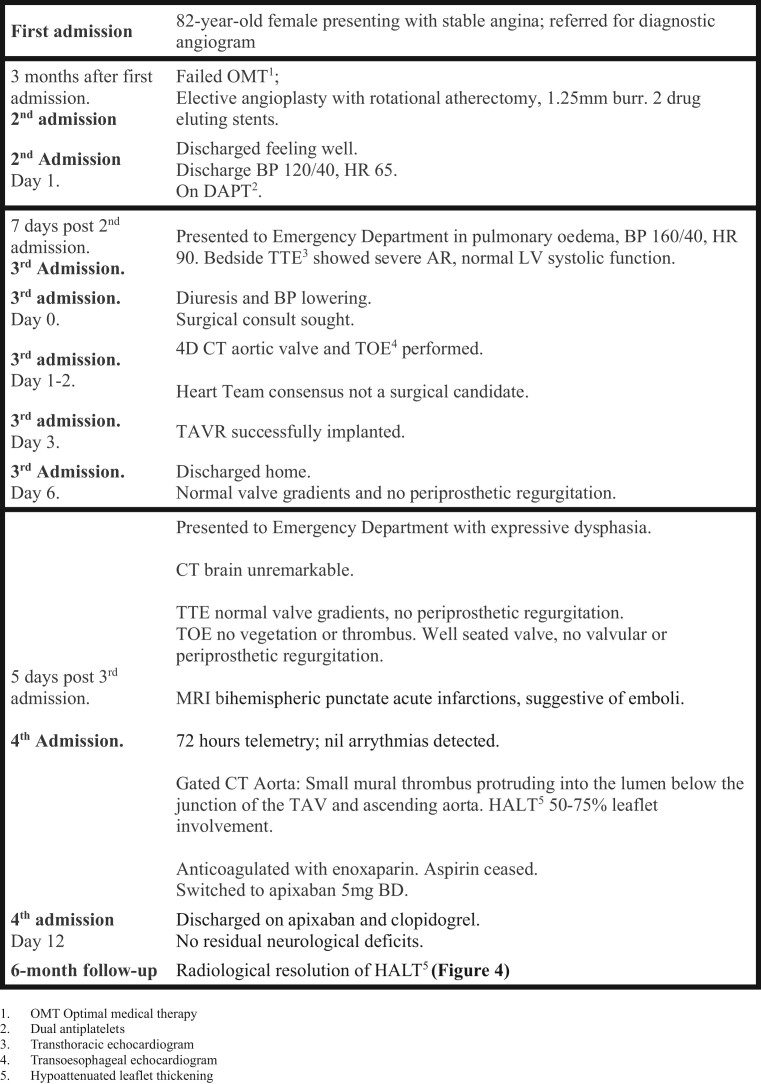


## Case report

**Figure 1:**
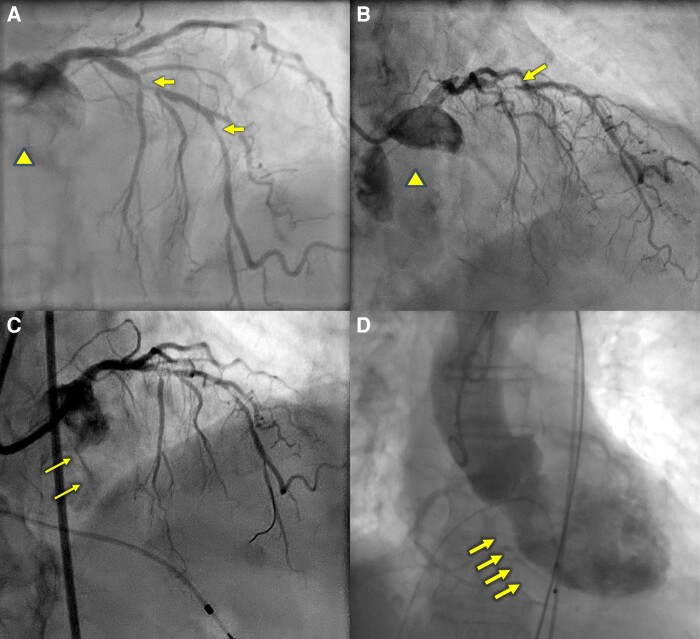
Angiogram, angioplasty procedure, and aortogram prior to TAVR. (*A* and *B*) Diagnostic coronary angiography demonstrating severe, calcific stenosis in the mid-left anterior descending artery (arrows) in two orthogonal views. Note the absence of AR as demonstrated by an absence of contrast refluxing into the left ventricle (triangles). (*C*) This is the first contrast injection following rotational atherectomy and before stenting. There is now contrast refluxing into the left ventricle. (*D*) Aortogram at the time of TAVR demonstrating severe AR.

Rotational atherectomy was performed for an 82-year-old female with a severely calcified left anterior descending (LAD) artery lesion (*Figure 1A* and *B*) presenting with increasing angina refractory to optimal medical therapy. The patient had a notable past medical history of hypertension, mild restrictive lung disease, and moderate 3A chronic kidney disease. She was on a maximally tolerated beta blocker, a long-acting nitrate, and multiple antihypertensives, including vasodilators. The patient had normal left and right ventricular size and systolic function and no significant valvular pathology. A 7 Fr Judkins Left 3.5 guide was used with a 1.25 mm burr. Five 15 s atherectomy runs with the 1.25 mm burr were performed. Despite poor guide support during atherectomy, the patient was successfully treated with two drug-eluting stents in the mid-LAD and was discharged on dual antiplatelets.

**Figure 2:**
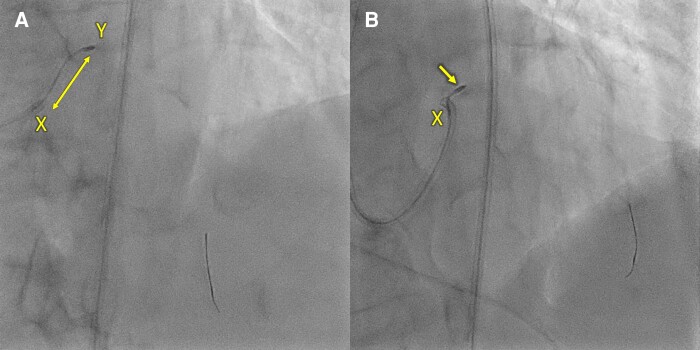
Rotational atherectomy to the LAD. (*A*) Significant redundancy in the coronary wire (arrow) with the guide disengaged in the aortic root (X). (*B*) The guide catheter is not co-axial with the left main (X). Rotational atherectomy commences after a forceful forward motion of the guide catheter, followed by the burr, against the left main ostia and left coronary cusp.

**Figure 3:**
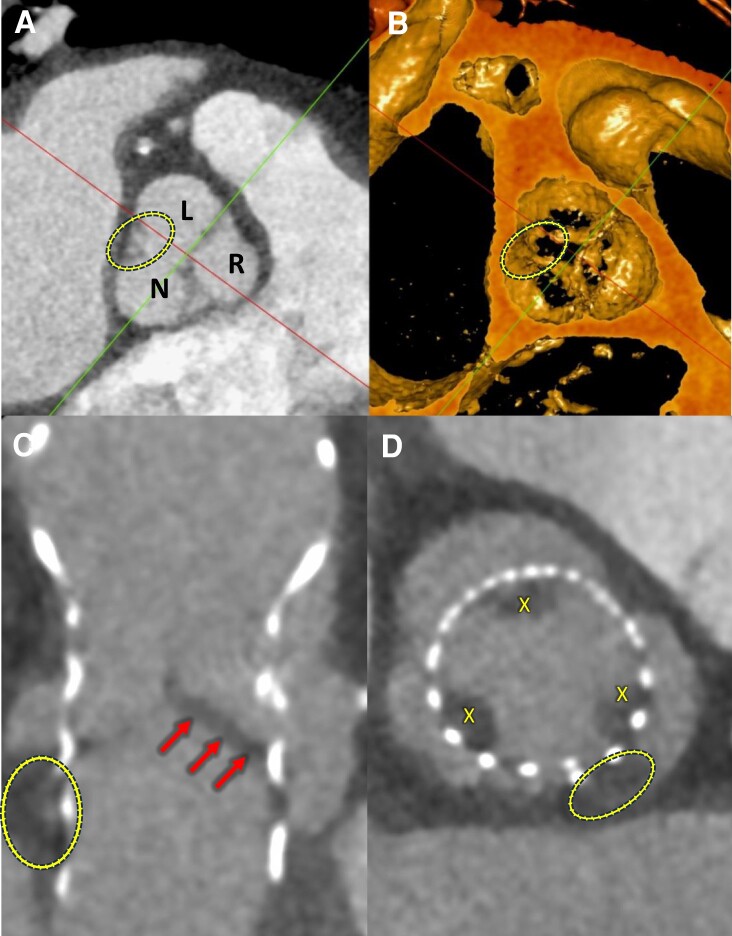
CT reconstructions of the aortic valve. (*A*) Hypoattenuation at the left coronary and non-coronary commissure (circle), suggestive of trauma to the commissure as seen on CT cardiac imaging after angioplasty, and also appreciated on (*B*). (*B*) 3D reconstruction of the aortic valve in cross section. (*C*) Dedicated post-TAVR CT after patient presented with a stroke. This demonstrates the pathognomonic meniscus sign indicating extensive leaflet thickening and hypoattenuating lesions (HALT) at the leaflets (arrows) and the sinuses (circle). (*D*) Extensive HALT of the leaflets (X) and sinuses (circle).

The patient presented one week later with progressive dyspnoea, culminating in her presentation to the Emergency Department with acute pulmonary oedema. Blood pressure was recorded at 160/40 mmHg and heart rate 90 beats per minute. Examination was consistent with pulmonary oedema. Bedside echocardiography demonstrated new severe AR with preserved LV systolic size and function (see Supplementary material online, *Video S1*). Upon review of the angioplasty procedure, AR had developed during the procedure following atherectomy, which was not immediately recognized (*Figure 1C* and *D*). The poor guide support and redundancy in the coronary wire resulted in forceful atherectomy beginning in the aortic root (see Supplementary material online, *Video S2*, *Figure 2A* and *B*), contributing to laceration of the aortic valve that was evident on echocardiography and CT, seen as hypoattenuation between the left and non-coronary commissure (*Figure 3A* and *B*). Given her age, pre-existing co-morbidities, frailty, and her presentation with overt heart failure, she was deemed high operative risk, and the Heart Team decision was made to treat with a TAVR. CT analysis demonstrated no aortic valve calcification, an annular area of 350 mm^2^ and perimeter of 67 mm. In a typical patient with such an annular size and severe aortic stenosis requiring TAVR, a 23 mm valve would be selected. Given the degree of AR, the increased stroke volume, and the absence of annular calcification, a significantly oversized 29 mm Evolut R valve was selected and successfully implanted with no procedural complications.

**Figure 4:**
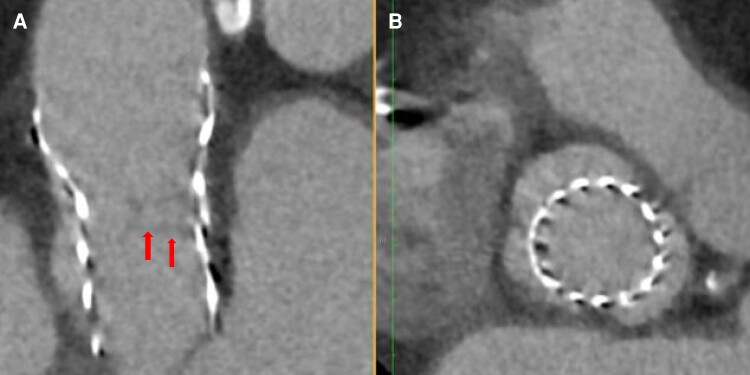
Repeat CT post-TAVR and after 6 months of anticoagulation showing resolution of HALT. (*A*) No evidence of ‘meniscus sign’ seen previously in *Figure 3C*. (*B*) Absence of HALT at the sinuses and leaflets of the aortic valve.

A week later, she presented with expressive dysphasia, and bi-hemispheric punctate acute infarcts were seen on MRI brain. A transoesphageal echocardiogram ruled out vegetations on the prosthesis, and no arrhythmias were detected on prolonged cardiac monitoring. Post-TAVR cardiac CT demonstrated extensive HALT and thrombus in the aortic sinuses (*Figure 3C* and *D*). She was anticoagulated with a non-vitamin-K-antagonist oral anticoagulant (NOAC) and clopidogrel and made full neurological recovery. Repeat cardiac imaging at 6 months demonstrated improvement with good TAVR function and complete resolution of leaflet thrombosis (*Figure 4A* and *B*). The patient reported complete resolution of angina, and there were no symptoms of heart failure.

## Discussion

Aortic valve laceration related to percutaneous coronary intervention is rare and to the best of our knowledge, has not been described during RA. It has been described in the literature as a complication of guide catheter engagement, coronary wires, and less commonly due to a hypersensitivity reaction, or repetitive mechanical injury with over-protruding ostial coronary stents, especially in the right coronary artery ostia.^1,2^

Rotational atherectomy is a useful adjunct in severely calcified coronary stenosis,^3,4^ however such equipment required to modify coronary calcium comes with a unique set of potentially serious complications. This case emphasizes important procedural and anatomical considerations when performing RA. Atherectomy should not be commenced in the aortic root without careful consideration of anatomical landmarks in orthogonal angiographic views. Co-axial guide engagement is paramount, particularly when approaching severely stenosed and distal lesions. In our case, the Judkins guide was not engaged in the left main. Atherectomy was commenced in the aortic root and as the severely calcified lesion in the mid-LAD was approached, there was redundancy in the coronary wire and on subsequent rota passes, the stored tension resulted in a forceful forward motion injuring the aortic valve leaflet/commissure (see Supplementary material online, *Video S2*).

Surgical aortic valve replacement (SAVR) is the treatment of choice for native pure AR^5^ and in the situation of traumatic or iatrogenic AR, surgical repair should be performed where possible. Mortality for patients who are not surgical candidates is high and TAVR for native pure AR poses unique anatomic and technical challenges.^6,7^ Amongst those challenges is the absence of valvular calcification that makes fluoroscopic visualization challenging and impairs sufficient anchoring of the valve increasing the potential for paravalvular leak.^6,7^ Patients with AR have increased stroke volume, and this haemodynamic state causes malposition and late valve migration. These issues can be mitigated by using a significantly oversized self-expanding valve in selected patients with the appropriate anatomy. This must be done in centres with expertise and whom expert Heart Teams have scrutinized multiple imaging modalities.

Lastly, this case highlights contributing factors, the diagnostic approach and management of HALT in the setting of post-TAVR stroke. There are several observations postulated for the increased incidence of HALT following TAVR, compared with SAVR. In TAVR, the native leaflets are pushed aside into the sinuses of Valsalva. This leads to changes in valve geometry, flow dynamics, and mechanical stress provoking an inflammatory and pro-thrombotic state.^8,9^ It is therefore not surprising that our patient with a significantly oversized prosthesis, implanted in the setting of illness and a pro-thrombotic state, developed HALT. Current guidelines prescribe a dedicated post-TAVR CT if there is a clinical suspicion for valve thrombosis or structural valve degeneration.^6^ Routine post-TAVR screening with CT and prophylactic anticoagulation has not yet been correlated with improved outcomes in randomized control trials.^9–11^

This case describes a rare and important complication and demonstrates our successful approach utilizing a multidisciplinary team to scrutinize multiple imaging modalities in the management of a complex, non-surgical patient.

## Supplementary Material

ytae226_Supplementary_Data

## Data Availability

The data underlying this article are available in the article and in its online supplementary material.

## Lead author biography

Dr Farrah Othman is an interventional cardiologist. She completed her cardiology training at Royal Prince Alfred Hospital in Sydney, prior to a 2-year high-volume interventional fellowship at the Fiona Stanley Hospital in Perth, Western Australia. During her fellowship, she was awarded the Geoff Mews ANZET Fellows Prize. She now works in Sydney, Australia, and has a special interest in complex coronary interventions, young persons, and women with cardiovascular disease and cardiometabolic diseases. Dr Othman is passionate about education, in particular simulation-based training in advanced life support and has been actively involved as a lecturer at University of Newcastle, Sydney, Australia, throughout her career.

## Supplementary material

**Consent:** Informed consent was granted from the patient in accordance with the Committee on Publication Ethics (COPE) recommendations.
