# Supplementary material for: Aortic valve laceration following rotational atherectomy: a case report
Source: Eur Heart J Case Rep. 2024 May 9;8(6):ytae226. doi: 10.1093/ehjcr/ytae226 (PMC11156196; doi:10.1093/ehjcr/ytae226)
Supplement: ytae226_Supplementary_Data [file ytae226_supplementary_data.zip › Supplementary videos caption.docx]

**Video 1** Bedside transthoracic echocardiography at second presentation: Parasternal long axis demonstrating normal LV size and function with new, severe aortic regurgitation.

**Video 2** Rotational atherectomy demonstrating insult to aortic valve: There is poor guide engagement, followed by forceful forward motion of the guide catheter with the burr into the left main ostia, where rotational atherectomy then commences into the LAD.
